# Impact of the village health center project on contraceptive behaviors in rural Jordan: a quasi-experimental difference-in-differences analysis

**DOI:** 10.1186/s12889-019-7637-9

**Published:** 2019-10-29

**Authors:** Makiko Komasawa, Motoyuki Yuasa, Yoshihisa Shirayama, Miho Sato, Yutaka Komasawa, Malak Alouri

**Affiliations:** 1 Department of Public Health, Faculty of Medicine, Juntendo University, 2-1-1 Hongo, Bunkyo-ku, Tokyo, 113-8421 Japan; 2 Faculty of International Liberal Arts, Juntendo University, 2-1-1 Hongo, Bunkyo-ku, Tokyo, 113-8421 Japan; 3 School of Tropical Medicine and Global Health, Nagasaki University, 1-12-4 Sakamoto, Nagasaki-city, 852-8102 Japan; 4 Atelier 514, 5-1-18 Kinuta, Setagaya-ku, Tokyo, 157-0073 Japan; 5 Directorate of Woman and Child Health, Ministry of Health, Prince Hamzah Bin Al Hussein Street, P.O. Box 940370, Amman, Jordan

**Keywords:** Evaluation, Impact, Difference-in-differences analysis, Family planning, Modern contraceptives, Community-based approach, Jordan, Japan International Cooperation Agency

## Abstract

**Background:**

Appropriate contraceptive use remains a major health challenge in rural Jordan. The Japan International Cooperation Agency implemented a project aimed at enhancing the capacity of village health centers (VHCs) to improve the quality and quantity of family planning (FP) services in rural Jordan in 2016–2018. Facility- and community-based approaches were integrated into the interventions. We evaluated the project’s impacts on contraceptive behaviors and the effectiveness of the two approaches.

**Methods:**

We used a difference-in-differences analysis based on the project baseline and endline surveys, and logistic regression analysis to assess associations between eight primary outcomes and three secondary outcomes (impacts). The unit of intervention was five target VHCs; the unit of analysis was currently married women of reproductive age (15–49 years) in five intervention and five control villages.

**Results:**

Overall, 2061 married women participated; 83.8% were in need of FP. Compared with the control villages, significant effects, ranging from + 0.4% points (pp) to + 11.5 pp., were observed in the intervention villages for six primary outcomes in these categories: increasing the use of FP services at VHCs, participation in health promotion activities, and changing the sources of reproductive health information. There was a trend toward improved secondary outcomes in the intervention villages, but no significant differences were observed between the intervention and control villages regarding modern contraceptive use (mCU; + 4.3 pp), traditional contraceptive use (tCU; − 0.5 pp), and spousal agreement on contraception (+ 5.1 pp). mCU was positively associated with five primary outcomes: obtaining contraceptives at VHCs [adjusted odds ratio (AOR) 3.44, 95% confidence interval (CI) 1.26–9.40], education sessions at VHC (AOR 7.41, 95% CI 1.60–34.39), health activities in communities (AOR 7.41, 95% CI 3.28–16.78), counseling by private doctor/clinic (AOR 0.62, 95% CI 0.40–0.97), and information gained through TV (AOR 0.50, 95% CI 0.32–0.76). Spousal agreement on contraception showed similar positive trends. tCU was associated only with TV.

**Conclusions:**

The project had impacts on increased mCU and husbands’ perception of contraception in rural Jordan. The integration of facility- and community-based approaches may be effective in shifting from tCU to mCU in other rural areas.

## Background

Over the past several decades, Jordan has faced issues of severe shortage of natural resources and rapid growth of population [1]. Furthermore, the massive influx of people from surrounding countries during this decade has become a major burden on the Jordanian health system [2]. The Ministry of Health (MOH) and the Higher Population Council, along with various partners in Jordan, have continuously implemented reproductive health projects, including family planning (FP) programs since the 1990s [2, 3]. Projects funded by the United States Agency for International Development (USAID) and the Japan International Cooperation Agency (JICA) have played major supporting roles in the field of reproductive health in Jordan [4, 5].

Due to the efforts of MOH and its partners, the contraceptive prevalence rate in Jordan increased from 40% in 1990 to 61% in 2012 [6]. However, this improvement was dependent on the use of traditional contraceptive methods (e.g. rhythm, withdrawal, and breastfeeding as a non-lactational amenorrhea method), and disparity remains between urban and rural areas [6]. The use of modern contraceptive methods [e.g. intrauterine devices (IUDs), pills, and male condoms, the top three contraceptive methods used in Jordan] has plateaued at 46% for currently married women of reproductive age (15–49 years) [6], which is lower than the world average of 58% and the average of other Muslim-majority countries in the middle eastern region, such as Egypt, Morocco, and Tunisia (59, 61, and 57%, respectively) [7]. These facts indicate that Jordan is still facing difficulties in transitioning from traditional to modern contraceptive methods. Using traditional methods or failure to use modern methods causes unintended pregnancies, leading to adverse health effects in both mothers and children [8–11]. Thus, appropriate FP practice remains a major health challenge in the rural areas of Jordan.

To improve the situation, JICA had implemented a technical cooperation project, entitled “Project for improvement of services at village health centers in rural host communities of Syrian refugees (the VHC project),” in collaboration with the Jordanian MOH from April 2016 to April 2018 [12]. This project primarily aimed at enhancing the capacity of village health centers (VHCs) to improve the quality and quantity of FP services and eventually accelerate the appropriate modern contraceptive use to reduce unintended pregnancies in rural northern Jordan. Under this project, two approaches were implemented: facility-based interventions for expanding quality FP service provision and community-based interventions for generating demands for modern contraceptive use.

Notably, few studies have evaluated the effectiveness of FP projects using a rigorous epidemiological method in Jordan [13]. Therefore, the present study aimed to evaluate the project’s impacts on contraceptive behaviors and determine the effectiveness of the two approaches in rural Jordan.

## Methods

### Project description

The Jordanian local health system comprises VHCs at the bottom and primary health centers (PHCs) and comprehensive health center (CHCs) as core primary healthcare facilities in the health directorate. The level of primary healthcare services provided by PHCs and CHCs is almost the same as that provided by general practitioners and midwives, including IUD insertions and removals. Generally, VHCs are located in rural areas where CHCs/PHCs are not available. These primary health care facilities provide services free of charge with the exception of some medications and IUD services. In principle, one nurse (various cadres from registered nurse to aid nurse) or midwife is assigned to one VHC apart from a part-time outreach doctor and a midwife from the upper CHCs/PHCs for 2–3 days per week. The minimum VHC services include provision of basic medications by the part-time doctor and more simple care by the nurse or midwife, such as vital sign monitoring, minor surgery, and provision of essential medication as per the doctor’s prescription. The current MOH regulation restricts the provision of FP services by trained midwives. However, there is a severe shortage of trained midwives and lack of essential equipment in rural areas that has led to the absence of FP services in rural communities.

To address these situations, the project team, together with MOH, decided to equip VHCs to enable them to provide FP services using the currently available staff. The project sites were Irbid, Mafraq, and Balqa health directorates. The 14 target VHCs (six in Irbid, six in Mafraq, and two in Balqa) were purposely selected by MOH and the project team on the basis of the location of these VHCs in rural areas that do not have access to advanced health facilities, as well as the availability of basic health personnel. Detailed selection criteria for the study sites are described in the study population subsection.

The facility-based interventions comprised five components: (i) providing a series of FP training for nurses or midwives in VHCs; (ii) conducting workshops for doctors and midwives who were periodically serving at VHCs; (iii) providing basic medical equipment; furniture; and information, education, and communication (IEC) materials required in FP services (i.e. FP counseling and providing pills and male condoms); (iv) conducting supervisory visits by maternal and child health supervisors from a local health directorate as well as MOH; and (v) updating the FP service manual for VHC staff. The community-based interventions also comprised five components: (i) supporting the establishment of a community health committee in each village; (ii) providing workshops to the committee members; (iii) encouraging committee members to make an action plan for the activities; (iv) monitoring their activities monthly; and (v) providing seed money for the first 4 months (a total of 100 Jordanian dinars, equivalent to approximately 140 US dollars as of October 2017). The actual period of the interventions was from October 2016 to January 2018 (13 months) for the facility-based interventions and from April 2017 to January 2018 (10 months) for the community-based interventions.

According to the project monitoring documents [12], four training sessions for the VHC health staff and three workshops for doctor/midwife were conducted. Consequently, the number of FP service users at six target VHCs in Irbid increased from 0 to 742 after a 13-month intervention. Regarding community activities, all villages established the community health committees, made action plans, and implemented various activities. Consequently, 103 health promotion events were conducted in six villages and the number of participants reached 2051 within a 10-month period. Activities conducted in each village varied from group educational sessions (e.g. at VHCs, schools, community-based associations, or village leaders’ houses), home-visits by VHC staff to free medical campaigns with a mobile clinic in collaboration with private companies.

### Study design

A difference-in-differences (DID) analysis was used to compare outcome changes overtime between the intervention and control groups to measure the impacts of interventions [14]. DID is one of the quasi-experimental designs that is frequently used for the estimation of causal relationships in settings where randomized control trials are infeasible or unethical [13, 15, 16]. Assuming that the outcome of the intervention group would follow “parallel trends” with the outcome of the control group over time in the absence of intervention, DID can help estimate the causal effects of the treatment [14].

The unit of intervention in this study was VHCs, and the unit of analysis was currently married women of reproductive age (15–49 years) who lived in the catchment areas of both the intervention and nonintervention VHCs. Primary outcomes were the direct benefits of the project, comprising three categories with eight indicators: (i) use of VHC with three indicators (FP counseling, obtaining contraceptives, and general counseling); (ii) participation in health promotion activities (education sessions at VHCs and health activities in communities); and (iii) sources of reproductive health information [counseling by VHC health staff or by private doctor/clinic, and information gained through TV (TV)]. Secondary outcomes were impacts on contraception behaviors and spousal’s perception of FP use: the percentage of modern contraceptive use (mCU), percentage of traditional contraceptive use (tCU), and percentage of spousal agreement on contraception.

A questionnaire was designed based on the Jordan Population and Family Health Survey 2012 (JPFHS 2012) [6], with three questions added from the survey conducted by the Jordan Communication, Advocacy, and Policy project funded by USAID [17]. The draft questionnaire was tested in Irbid villages, which were neither intervention nor control areas, and was modified for suitability to the local participants. Questions included participants’ basic characteristics, household conditions, contraceptive behaviors, VHC use, participation in health promotion activities, and sources of reproductive health information.

A field survey was conducted from September to October 2016 for baseline and from January to February 2018 for endline. Trained female interviewers visited households and interviewed the eligible women using the structured questionnaire. Written informed consents were obtained from all participants. For married adolescents aged 15–17 years, in addition to the written informed consents, verbal consents were obtained from their husbands, parents-in-law, or other legal guardians during initial contact with the participants.

### Study population

**Fig. 1:**
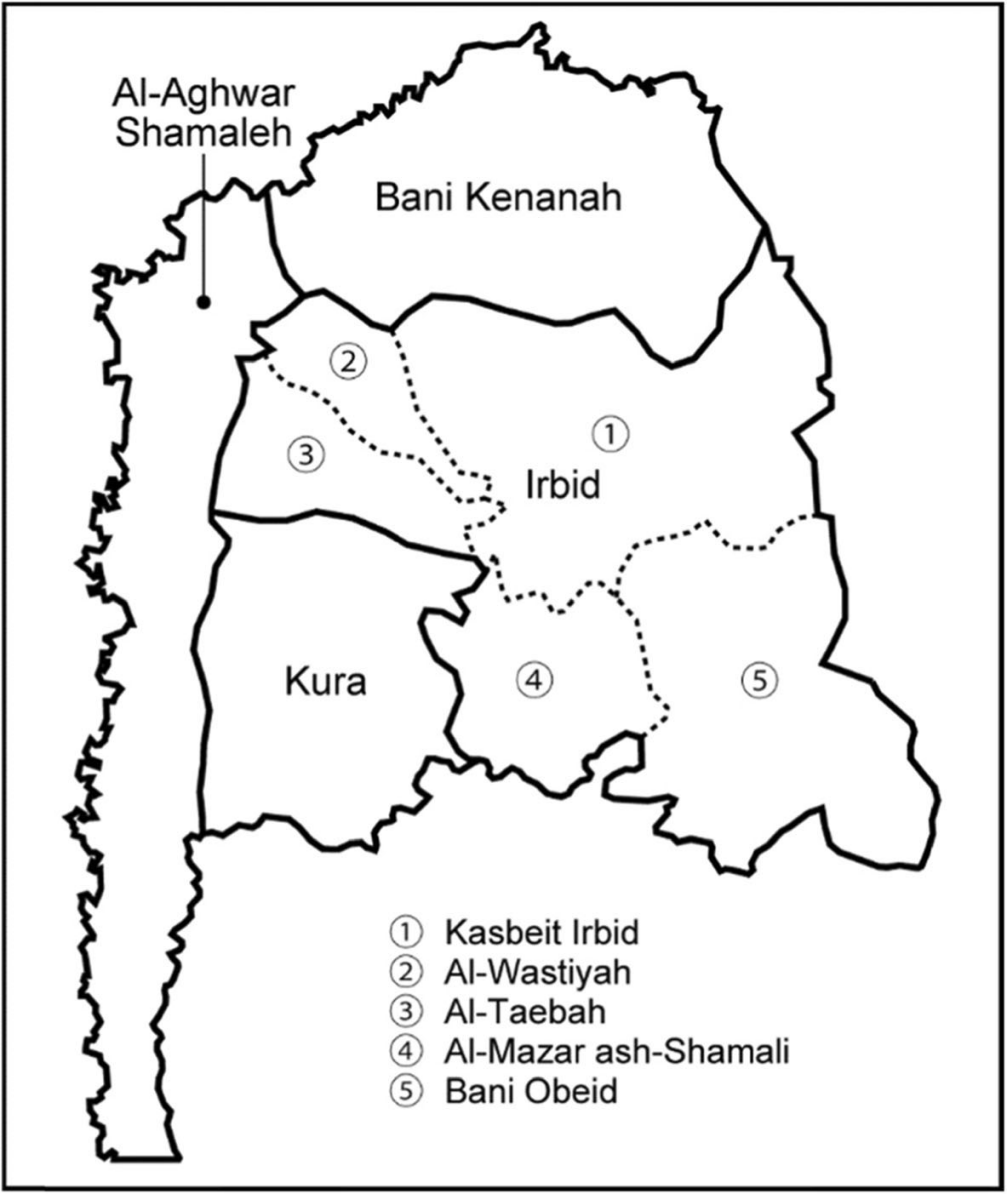
Map of Irbid health directorate. Footnote: The bold lines indicate the boundary of four health districts (Irbid, Kura, Al-Aghwar Shamaleh, and Bani Kenanah), and the dotted lines indicate the boundary of five districts in the Irbid health district (Kasbeit Irbid, Al-Wastiyah, Al-Taebah, Al-Mazar ash-Shamail and Bani Obeid)

Among the three project-target directorates, the study team selected Irbid health directorate as the study area because the other two health directorates had previously received similar interventions by other programs. Irbid health directorate has a population of 1.8 million [18]; it is located 100 km north of Amman, sharing a border with Syria. Generally, the upper northern and western parts of the Irbid health directorate are rural and remote areas where people experience difficulties in accessing health facilities, whereas the southern parts are urban. The project team selected one intervention VHC from each health district (Fig. 1). Regarding Irbid health district, there are five districts; the project team selected one VHC in the Taebah district and two in the northern part of the Kasbeit Irbid district because of the large area and population of the latter. Among the six intervention VHCs, the study team excluded one VHC in the Kasbeit Irbid because two VHCs were closely located. With respect to the control VHCs, the study team chose a VHC to match an intervention VHC from the same health district on the basis of similar demographic and economic characteristics. However, because the Kura health district has only one VHC, its control VHC was chosen from the Al-Wastiyah district.

### Sampling design

To determine the sample size of the catchment population in each VHC, the probability proportionate to the population size was applied, based on the household frame of the 2015 Jordan Census [18]. In each village, participating households were selected by systematic random sampling. If more than one eligible woman was available in one household, the interviewer randomly selected one participant. In case of absence or unavailability of eligible woman on the second visit, the household was replaced by a neighboring house.

We estimated an mCU of 40% in the intervention area at baseline on the basis of data of JPFHS 2012 [6], and the result obtained using the following formula indicated that the required minimum sample size was at least 369:

$$ N=\frac{4{Za}^2\times P\left(1-P\right)}{W^2} $$

*N* = minimum sample size required for systematic random sampling

Zα = standard normal deviation at 95% confidence level (1.96)

*P* = estimated mCU in a study area (40%)

*W* = confidence interval

Considering the stratification effect, invalid questionnaires, and prevalence of currently infecund women, the sample size was increased to 500 for both the intervention and control groups. Thus, we intended to recruit a total of 1000 participants for both baseline and endline surveys.

### Data analysis

First, the basic characteristics of participants were compared between the intervention and control groups at baseline and endline. Second, the DID analysis was conducted using the eight primary outcome indicators at both baseline and endline between the two groups. To obtain mCU and tCU in the impacts, the number of women in need of FP (currently fecund women) was identified. Third, the DID analysis was used to evaluate the impacts (mCU, tCU, and spousal agreement on contraception). Finally, logistic regression analysis (reference was “no”) was performed to examine the association between the eight primary outcomes and the three impacts separately at endline point. *P* <  0.05 was considered statistically significant. STATA version 15 (STATA Corporation, Texas, USA) was used for DID analysis and SPSS version 25 (IBM, Chicago, USA) for other statistical analyses.

## Results

### Participant characteristics

In total, 510 and 509 women at baseline and 508 and 534 at endline were enrolled in the intervention and control groups, respectively. There were no significant differences in the sociodemographic characteristics between the intervention and control groups at both baseline and endline (Tables 1 and 2). The number of women in need of FP (fecund women) in the intervention and control groups was 434 and 432 at baseline and 426 and 435 at endline, respectively.

**Table 1:** Basic characteristics of participants of intervention and control groups at baseline

Variable	Intervention group		Control group		*P*-value
	(*N* = 510)		(*N* = 509)		
	*n*	(%)	*n*	(%)	
Age (mean [SD])	34.4	[7.86]	34.4	[8.18]	0.91^a^
Nationality					
Jordan	483	(94.7)	490	(96.3)	0.32^b^
Syrian	26	(5.1)	17	(3.3)	
Others	1	(0.2)	2	(0.4)	
Years of school attendance (years)					
No education	5	(1.2)	2	(0.9)	0.68^b^
1–10	141	(27.4)	135	(29.8)	
11–12	219	(41.4)	225	(40.3)	
13+	145	(30.0)	146	(29.0)	
Age at first marriage (mean [SD])	21.1	[4.32]	21.6	[4.80]	0.06^a^
Number of children (mean [SD])	3.5	[2.18]	3.4	[2.04]	0.28^a^
Years at current residence (mean [SD])	9.1	[7.89]	8.4	[7.70]	0.14^a^
Monthly household income^c^ (mean [SD])	380.3	[175.92]	382.2	[185.25]	0.87^a^
(Medium)	(350.0)		(350.0)		

*SD* standard deviation

^a^*t*-test

^b^Chi-square test

^c^Jordan dinar

**Table 2:** Basic characteristics of participants of intervention and control groups at endline

Variable	Intervention group		Control group		*P-*value
	(*N* = 508)		(*N* = 534)		
	*n*	(%)	*n*	(%)	
Age (mean [SD])	34.5	[7.97]	34.4	[8.62]	0.79^a^
Nationality					
Jordan	482	(94.9)	507	(94.9)	0.24^b^
Syrian	25	(4.9)	22	(4.1)	
Others	1	(0.2)	5	(0.9)	
Schooling (years)					
No education	6	(1.2)	5	(0.9)	0.84^b^
1–10	139	(27.4)	159	(29.8)	
11–12	210	(41.4)	215	(40.3)	
13+	152	(30.0)	155	(29.0)	
Age at first marriage (mean [SD])	21.0	[4.35]	21.2	[4.46]	0.44^a^
Number of children (mean [SD])	3.5	[2.13]	3.3	[1.96]	0.08^a^
Years at current residence (mean [SD])	9.2	[7.89]	9.4	[8.05]	0.72^a^
Monthly household income^c^ (mean [SD])	391.8	[204.31]	374.8	[177.73]	0.16^a^
(Medium)	(350.0)		(350.0)		

*SD* standard deviation

^a^*t*-test

^b^Chi-square test

^c^Jordan dinar

### Effects of intervention on primary outcomes

Table 3 presents the DID analysis results for primary outcomes. Regarding the use of services at VHCs, the intervention effects for the three indicators FP counseling, obtaining contraceptives, and general counseling increased by + 4.1 percentage points (pp.), + 7.6 pp., and + 4.9 pp., with *P* = 0.004, < 0.001, and 0.005, respectively. The two indicators of participation in health promotion activities showed substantial effects between baseline and endline: education session at VHCs and community health activities had intervention effects of + 11.5 pp. and + 8.1 pp., respectively, with *P* <  0.001 for both. Regarding the sources of reproductive health information, counseling by VHC staff showed an upward trend, whereas counseling by private doctor/clinic and TV exhibited a downward trend in both the intervention and control groups. Considering changes in the control group as counterfeit counterfactuals, six of the eight primary indicators in the intervention group showed expected effects at significant levels.

**Table 3:** Effects of the intervention on primary outcomes from the DID analysis

Variable	Intervention group (%)			Control group (%)			Difference	Assumption^a^	DID	*P*-value
	Baseline	Endline	*P*-value	Baseline	Endline	*P*-value	e	f	(e – f)	
	(*N* = 510)	(*N* = 508)		(*N* = 509)	(*N* = 534)					
	a	b		c	d		b − a	d − c		
Use of VHCs’ services										
FP counseling	0.2	4.9	0.001	2.4	3.0	0.52	4.7	0.6	4.1	0.004
Obtaining contraceptive	1.4	8.9	< 0.001	2.2	2.1	0.91	7.5	− 0.1	7.6	< 0.001
General counseling	1.4	6.3	< 0.001	4.7	4.7	0.98	4.9	0.0	4.9	0.005
Participation in health promotion activities										
Education sessions at VHCs	2.9	18.9	< 0.001	2.4	6.9	0.54	16.0	4.5	11.5	< 0.001
Health activities in communities	0.8	8.7	< 0.001	0.4	0.2	< 0.001	7.9	− 0.2	8.1	< 0.001
Source of reproductive health information										
Counseling by VHC staff	5.5	14.2	< 0.001	3.1	6.2	0.02	8.7	3.1	5.6	0.012
Counseling by private doctor/clinic	42.0	35.6	0.04	43.0	34.5	0.01	− 6.4	− 8.5	2.1	0.639
TV	65.9	52.0	< 0.001	68.2	53.9	< 0.001	− 13.9	− 14.3	0.4	0.963

*DID* difference-in-differences, *VHCs* village health centers, *FP* family planning

^a^Counterfactual assumption

### Effects of intervention on secondary outcomes

Table 4 shows the DID estimates of the secondary outcomes (impacts). mCU increased from 47.0% at baseline to 51.9% at endline in the intervention group and from 48.8% to 49.4% in the control group, resulting in + 4.3 pp. effect (*P* = 0.376). Similarly, the effects of tCU and spousal agreement on contraception were − 0.5 pp. (*P* = 0.901) and + 5.1 pp. (*P* = 0.057), respectively. Expected effects were observed after controlling, although there was no significant difference in the secondary outcomes between the two groups.

**Table 4:** Effects of the intervention on secondary outcomes from the DID analysis

Variable	Intervention group (%)			Control group (%)			Difference	Assumption^a^	DID	*P-*value
	Baseline	Endline	*P*-value	Baseline	Endline	*P*-value	e	f	(e - f)	
	*(N* = 434)	*(N* = 426)		*(N* = 432)	*(N* = 435)					
	a	b		c	d		b − a	d − c		
Modern contraceptive use	47.0	51.9	0.15	48.8	49.4	0.86	4.9	0.6	4.3	0.376
Traditional contraceptive use	23.7	25.4	0.58	22.2	24.4	0.46	1.7	2.2	− 0.5	0.901
Spousal agreement on contraception	86.5	92.5	0.001	88.6	89.5	0.07	6.0	0.9	5.1	0.057

*DID* difference-in-differences

^a^Counterfactual assumption

### Association between primary and secondary outcomes

Table 5 summarizes the results on the association between the three secondary outcomes (mCU, tCU, and spousal agreement on contraception) and the eight primary outcomes at endline using the logistic regression analysis separately. mCU showed positive association with five primary outcomes with a significant difference: obtaining contraceptives at VHCs [adjusted odds ratio (AOR) 3.44, 95% confidence interval (CI) 1.26–9.40], education sessions at VHC (AOR 7.41, 95% CI 1.60–34.39), health activities in communities (AOR 7.41, 95% CI 3.28–16.78), counseling by private doctor/clinic (AOR 0.62, 95% CI 0.40–0.97), and TV (AOR 0.50, 95% CI 0.32–0.76). Conversely, tCU was associated with only one primary outcome: TV (AOR 0.40, 95% CI 0.21–0.75). Spousal agreement on contraception was influenced by six primary outcomes: FP counseling at VHC (AOR 12.01, 95% CI 1.49–96.57), obtaining contraceptives at VHC (AOR 3.28, 95% CI 1.35–8.00), education sessions at VHC (AOR 6.72, 95% CI 2.28–19.84), health activities in communities (AOR 6.22, 95% CI 3.45–11.21), counseling by private doctor/clinic (AOR 0.71, 95% CI 0.53–0.95), and TV (AOR 0.48, 95% CI 0.36–0.65).

**Table 5:** Factors associated with three secondary outcomes (impacts) by multivariate logistic regression analysis

Variable	Modern contraceptive use	Traditional contraceptive use	Spousal agreement on contraception
	(*N* = 427) Adjusted odds ratio (95% CI)	(*N* = 212) Adjusted odds ratio (95% CI)	(*N* = 911) Adjusted odds ratio (95% CI)
Use of VHCs’ services			
FP counseling	5.01 (0.55–45.82)	–	12.01 (1.49–96.57)*
Obtaining contraceptives	3.44 (1.26–9.40)*	2.33 (0.15–37.38)	3.28 (1.35–8.00)**
General counseling	2.26 (0.54–9.45)	1.99 (0.45–8.69)	2.43 (0.98–6.07)
Participation in health promotion activities			
Education sessions at VHC	7.41 (1.60–34.39)**	–	6.72 (2.28–19.84)**
Health activities in communities	7.41 (3.28–16.78)***	–	6.22 (3.45–11.21)***
Source of reproductive health information			
Counseling by VHC staff	1.25 (0.53–2.94)	1.70 (0.53–5.48)	1.45 (0.83–2.55)
Counseling by private doctor/clinic	0.62 (0.40–0.97)*	0.53 (0.28–1.00)	0.71 (0.53–0.95)*
TV	0.50 (0.32–0.76)**	0.40 (0.21–0.75)*	0.48 (0.36–0.65)***

*CI* confidence interval, *VHCs* village health centers, *FP* family planning; −, not presented due to the small numbers of prevalence at base line time

*P < 0.05, **P < 0.01, ***P < 0.001

## Discussion

To the best of our knowledge, this is the first study to evaluate the impacts of the project on FP behavior changes in Jordan using a rigorous method. The DID analysis revealed that the project interventions increased the utilization of FP services at VHCs and participation in health promotion activities. The estimated effects also indicated an increase in modern contraceptive use and spousal acceptance of contraception. The association between the primary indicators and secondary indicators (impacts) shows that integrating the facility- and community-based approaches may contribute to the emergence of these impacts.

The results obtained for the primary outcomes showed that the project interventions of expanding FP services at VHCs promoted the utilization of FP services at VHCs. This result may be explained by two reasons. First, the project began to provide free FP services (i.e. counseling and providing pills and male condoms) at VHCs, which were easily accessible for local women whose primary transportation means was walking: 85.1% of all women who used VHCs in the previous year walked to the facility according to the information collected in the endline survey. Second, the quality of services at VHCs may have visibly improved (e.g. better attitude of trained health personnel and availability of standardized counseling using updated IEC materials and other tools for FP counseling in VHCs). This was supported by the fact that 47.9% of study participants who used VHCs in the past year recognized improvement in the services within 1 year at endline: 82.7% participants reported improvements regarding increased variety of services, 35.1% reported improvement in hardware setting, 34.6% reported improvement in IEC materials, and 51.4% reported a better attitude of nurses or midwives. Moreover, participation in health promotion activities substantially increased. Although some local women from the control areas may have attended the health promotion activities in intervention areas, it did not result in overvaluation of the project effect. These primary outcomes may induce impacts regarding FP behavior and perception changes.

There have been changes in the trends in sources of reproductive health information: an increase in counseling by VHC staff, whereas a decrease in counseling by private doctor/clinic and FP information through TV. These results indicate that the project interventions led to a shift from gaining information through private facilities or mass media to face-to-face counseling by local VHCs. This tendency was inconsistent with the findings of previous studies. First, a Jordanian study reported that Jordanian women in urban areas relied more on private doctor/clinic for using modern contraceptive services [11]. Second, it is believed that mass media has been effectively delivering FP messages for several decades worldwide [8, 19]; however, the effect is observed only when mass media is used through programmatic approaches, such as creating entertainment (e.g. soap opera), combining with social marketing, or developing interpersonal communication mechanisms [13]. Our results indicated that in rural Jordan, free, interpersonal counseling at walking distance was more appreciated than that provided in private facilities or provided through mass media as a source of FP information.

The expected impacts of the project were contraceptive behavior changes and acceptance. The DID estimates suggested that the project may have increased mCU (+ 4.3 pp.) and decreased tCU (− 0.5 pp.) within the 13 months of its implementation; however, these impacts were not significantly different between the intervention and control groups. One possible reason behind the moderate increase in mCU was that VHCs did not provide IUD services, which was the most popular modern method (used by 21.3% of all married women aged 15–49 years) in Jordan [6], due to the lack of equipment and skilled personnel. From a different viewpoint, Brown et al. [20] estimated that the annual modern contraceptive prevalence growth rate was 2.8% for high growth levels between 2016 and 2020 worldwide. In comparison with this estimated rate, the growth rate in our intervention group in 13 months was 4.9%, whereas this rate in the control group was 0.6%. The estimated effect of spousal agreement on contraception was also positive, indicating that the project interventions may have raised the husbands’ awareness regarding the importance of FP and encouraged couples’ attitude and practice toward the use of modern methods [21–23].

To assess the effectiveness of integrating the facility- and community-based approaches, we investigated the association between the impacts and primary outcomes. mCU was positively associated with obtaining contraceptives at VHCs and two kinds of health promotion activities and negatively associated with counseling by private doctor/clinic and TV. The spousal agreement on contraception showed similar trends, with additional positive association with FP counseling at VHCs. These results suggest that in a facility-based intervention, free contraceptives at easily accessible delivery points, with sufficient information through qualified interpersonal counseling, could encourage rural wives to communicate with their husbands and easily convince them for a broad consensus [13, 21]. The community-based intervention in this project was to start establishing community health committees comprising local leaders, both male and female, in each community; subsequently, appropriate FP messages would be delivered by health professionals and local networks to male decision makers in communities or at home. This is in line with a previous study in the Islamic world [24], which illustrated that a community-based approach is effective in addressing the social barriers and lack of access to information concerning FP and in increasing the adoption of modern contraception. Contrarily, tCU showed a weak association with the primary outcomes, which might be because the project did not involve direct intervention of tCU. These findings imply that the positive impacts are attributable to facility- and community-based interventions, in line with the common findings of previous studies [8, 14, 25, 26].

Generally, to produce behavioral outcomes of FP, five essential elements are required: (i) adequate supply of safe and effective methods, (ii) quality of care, (iii) conscientious rights of both service providers and users, (iv) community engagement in introducing the FP concept, and (v) commitments to laws and policies [13, 27, 28]. The facility-based approach in this project covered the elements from (i) to (iii) and the community-based approach coped with (iv). In addition, MOH as a co-implementer committed to integrate the project approaches into the new governmental strategy [29]. Roudi-Fahimi F et al. [27] also insisted that a combination of supply-side and demand-side interventions was necessary to meet the FP demands in the Arab countries. The project in the present study considered these aspects and was designed to obtain synergistic effects of integrating the facility- and community-based approaches by strengthening VHCs. The integration of two approaches may contribute to produce visible outcomes within a relatively short duration of implementation. However, the extent of each element’s contribution, such as providing contraceptives alone, quality counseling, women’s convenience to visit VHCs, local leaders’ commitment, and local leaders’ influence on the husbands’ attitude, toward the impacts and degree of synergy effects among elements was not measured.

There are some limitations to our study. First, the main limitation of this study is that we used the quasi-experimental design instead of the randomized controlled design. The DID analysis does not perfectly eliminate unobserved biases, which means that the estimated results may have been biased or invalid [13]. Despite this limitation, we believe that the DID analysis was best suited to measure the impact of government-led projects because performing evaluation of projects of this nature in a purely experimental manner is difficult due to both ethnical and practical issues [13, 14]. Second, seasonally mobile people were observed in the study areas between summer (July–September) and winter (November–February). Moreover, during the endline survey in January, several women were away from home during daytime because they were harvesting olives as their part-time job, which may have caused selection bias. However, we believe that the selection bias may not have affected the comparison much because such women were present in both the intervention and control populations. Third, our study did not include the factors related to the knowledge of contraceptives because the previous studies have reported that the knowledge of contraceptives was universal and not a barrier to practice FP in Jordan [6, 17]. However, sufficient knowledge of modern methods, including fears of adverse health risks, side effects, and future childbearing, called for increasing attention as crucial factors related to the nonuse of modern methods [26, 30]. Therefore, further studies are necessary to identify the extent of knowledge of modern methods and how to shift from tCU to mCU. Fourth, it is important to assess the project sustainability after the intervention is completed. This study did not examine whether the impact will sustain after the end of the project and did not predict the future impacts. Thus, further studies are required to address these limitations.

## Conclusions

The VHC project exerted the expected effects on the use of FP service at VHCs and participation in health promotion activities as well as the impacts on increasing mCU and husbands’ perception of contraception. The findings also indicated that the integration of the two approaches, facility- and community-based approaches, may have resulted in these impacts in the rural settings. This study will provide valuable insights for designing future FP projects not only in Jordan but also in rural areas of other countries that are still in the transitional phase from traditional to modern contraception.

## Data Availability

The data that support the findings of this study are available from the Japan International Cooperation Agency, but restrictions apply to the availability of these data, which were used under license for the current study. However, data are available from the authors upon reasonable request and with permission from Japan International Cooperation Agency.

## Abbreviations

AOR: Adjusted odds ratio
CHCs: Comprehensive health centers
CI: Confidence interval
DID: Difference-in-differences
FP: Family planning
IEC: Information, education and communication
IUD: Intrauterine device
JICA: Japan International Cooperation Agency
JPFHS: Jordan Population and Family Health Survey
mCU: percentage of modern contraceptive use
MOH: Ministry of Health
PHCs: Primary health centers
pp.: Percent point
tCU: Percentage of traditional contraceptive use
USAID: United States Agency for International Development
VHC: Village health center
